# Professional Pride

**Published:** 1874-02

**Authors:** F. A. Badger


### PROFESSIONAL PRIDE.

BY F. A. BADGER.

The profession of dentistry *per se* will always honor the dentist, though he may not honor the profession. It is a lamentable thought that many dentists seek throughout life the empty honorary title of M. D., and labor and croak assiduously to have dentistry fully acknowledged as a specialty of medicine. If their herculean efforts were devoted to a thorough knowledge of those portions of anatomy and physiology which pertain to the teeth and their adjacent structures, together with their pathological conditions, so that they might scientifically address therapeutical agents for their permanent relief and healthfulness, much more good would be accomplished, and the greatest stride yet made for reaching the goal of their ambition, the Eldorado of their happiness—a full recognition as a specialty of medicine would be accomplished.

My father, the la e Dr. F. H. Badger, who practiced dentistry many years in Nashville, once told me, if one wishes to be *considered* great, he must *be* great; if we would be the peers of the followers of Galen, and desire their recognition as such, we must *be deserving.* There are those in our profession who fear their wisdom, excellence and genius are not appreciated; they fear they will die unwept, unhonored and unsung. The world is proverbially slow to award praise; but it is certain to bestow it upon those who are worthy. If we achieve excellence, it is a happy thought, that death and the grave can not rob us of our laurels; but Fame's beantiful wreath will grow brighter, and each year will add to its scintillations.

As a dentist, I am proud of my profession, *per se*, recognized or unrecognized by the medical fraternity. Away with this itching desire for medical favors! Have we not men in the profession who would honor any profession? Have we not men who would cause our professional bark

> "To ride the waters like a thing of life,
> And seem to dare the elements to strife"?

And even though the tempests prevail, and angry waters lash its timbers, we have men fully competent for any emergency, and who would totally and unequivocally reject the patronizing council of an M. D., especially when it often occurs that the said M. D. has no chance for immortality except the halo of imaginary glory that glitters around his title.

In this government men are esteemed in proportion to the amount of good they do; their reward is in proportion to their ability, industry and integrity: while in Europe they may fritter away their life in idleness, and still be esteemed —if they have riches and a noble title.

